# Suspected autochthonous *Thelazia callipaeda* infection in a dog in northern Germany

**DOI:** 10.1007/s00436-020-06920-z

**Published:** 2020-10-13

**Authors:** Sophia L. Lebedewa, Kevin Tkocz, Peter-Henning Clausen, Ard M. Nijhof

**Affiliations:** 1 Institute for Parasitology and Tropical Veterinary Medicine, Department of Veterinary Medicine, Freie Universität Berlin, Berlin, Germany; 2 Veterinary Practice‚ Im Kalten Tale, Wolfenbüttel, Germany

**Keywords:** *Thelazia callipaeda*, Germany, Cytochrome c oxidase I, Zoonosis

## Abstract

A 12-year old Elo dog was presented with recurring symptoms of conjunctivitis in November 2019. A single whitish nematode was found upon inspection of the eye and identified as a *Thelazia callipaeda* male. The morphological identification of the eye worm was supported by analysis of a partial cytochrome c oxidase I (*cox1*) gene sequence. The dog lived in Lower Saxony, northwestern Germany, and had not visited regions known to be endemic for *T. callipaeda.* This suggests that a local transmission cycle of this zoonotic nematode may exist in Germany.

## Introduction

*Thelazia callipaeda* (Spirurida, Thelaziidae) are nematodes that occur in the eyes of carnivores, particularly dogs and foxes, as well as lagomorphs and sometimes humans (Gama et al. 2016; Otranto and Deplazes 2019). The parasite was first described from Asia in 1910 (Railliet and Henry 1910), where it is widespread. The first case of canine thelaziosis in Europe was described in Italy in the late 1980s (Rossi and Bertaglia 1989), and the distribution of this ’oriental eye worm’ has since extended to larger parts of Europe, with enzootic areas in southern Europe (do Vale et al. 2019).

Adult *T. callipaeda* lives in the orbital cavity of the definitive host, where they reach sexually maturity after about 3–4 weeks post-infection and can survive for more than 1 year (Faust 1928; Kozlov 1962; Anderson 2000; Otranto et al. 2004). First-stage larvae (L1) produced by the larviparous female eye worm are released in the lachrymal secretions. The non-biting fruit fly *Phortica variegata* that feed on these secretions can become infected with the L1 and act as intermediate hosts and vector for this nematode in Europe (Otranto and Dantas-Torres 2015). Under natural conditions, male *P. variegata* flies appear to be the main vectors for *T. callipaeda*, as female *P. variegata* flies prefer fruit as their food source, whereas males show a more zoophilic behaviour (Otranto et al. 2006). In male *P. variegata*, the L1 migrates from the crop to the testes where it develops to the infective L3 stage. The L3 migrates back to the proboscis from where it is transmitted to the definitive host when the fly feeds on ocular secretions again (Otranto et al. 2005). Further development to the adult stage takes place on the surface of the eye.

Infections with *T. callipaeda* may be subclinical but can also cause symptoms of conjunctivitis such as lacrimation and pruritus and, in severe cases, keratitis and ulceration (Marino et al. 2020).

Canine thelaziosis caused by *T. callipaeda* has previously been reported from Germany and was associated with travel to an endemic region in Italy (Hermosilla et al. 2004) or originated from a region close to the French border in southern Germany (Magnis et al. 2010). We here report on a case of canine thelaziosis in a dog from northwestern Germany who had not entered regions known to be endemic for *T. callipaeda*, making a local transmission likely.

## Material and methods

### Animal

A 12-year-old, female Elo dog living in Lower Saxony, northwestern Germany, was presented to a veterinarian in November 2019 for a check-up for distichiasis since it showed recurring unilateral symptoms of a slight conjunctivitis and pruritus. The symptoms had first appeared early August 2019, and the owner initially treated it with homoeopathic eye drops, which provided only short-term relief. During the clinical examination, a single whitish worm was detected in the left eye. The dog was subsequently treated with a spot-on product containing moxidectin and imidacloprid (Prinovox®, Virbac). Clinical symptoms were not observed during clinical examinations conducted at 5 days and 2 months post-treatment. The dog had been treated in July 2019 with a spot-on product containing permethrin and imidacloprid (Advantix®, Bayer Animal Health) for tick bite prevention.

The dog had crossed the German-French border near Saarbrücken once for a single day in April 2017 and had travelled to Denmark and Sweden in August 2019, after the conjunctivitis had appeared for the first time.

### Morphological identification

The adult nematode was initially stored in Ringer’s solution and submitted to the Institute for Parasitology and Tropical Veterinary Medicine, where it was stored in 70% ethanol before examination using a Stemi 2000-C microscope (Carl Zeiss, Jena, Germany).

### Molecular identification

A ~ 700-bp fragment of the cytochrome c oxidase I (*cox1*) gene was amplified using primers COI_Nema_fw (5′-GAAGTTCTAATCATAARGATATTGG-3′) and COI_Nema_Rv (5′-ACCTCAGGATGACCAAAAAAYCAA-3′) (Duscher et al. 2015). PCR reactions (20 μl) were performed with 0.2 mM dNTPs, 250 nM of each primer, 0.1 U Phusion Hot Start II High-Fidelity DNA polymerase (Thermo Scientific) and 2 μl template DNA in 1× HF buffer under the following cycling conditions: an initial denaturation step at 98 °C for 30 s, followed by 40 cycles of 98 °C for 10 s, 55 °C for 30 s and 72 °C for 30 s and a final elongation step at 72 °C for 5 min. The amplicons were subsequently purified using DNA Clean and Concentrator kit (Zymo Research), cloned using the StrataClone Blunt PCR Cloning Kit (Agilent) using the manufacturer’s instructions. Plasmids containing an insert of the expected size were sequenced in both directions (LGC Genomics, Berlin, Germany). The resulting sequence was edited using BioEdit (v7.2.5).

## Results

**Fig. 1:**
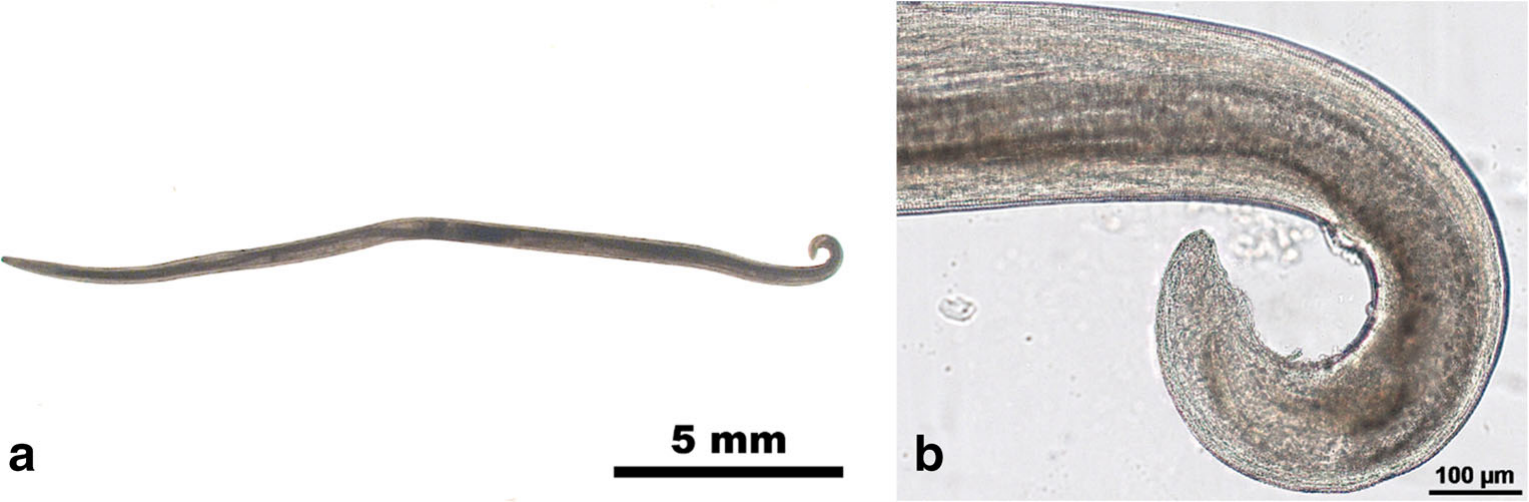
The *Thelazia callipaeda* male found in the eye (**a**), with a detailed view of the curved posterior end (**b**). Photographs by K. Seidl, Freie Universität Berlin

The nematode was morphologically identified as a male *Thelazia callipaeda* worm (Fig. 1).

This morphological identification was confirmed by amplification and subsequent sequencing of a partial fragment of the *cox1* gene sequence. This sequence was 100% (683/683 nt) identical to the *cox1* sequence of a complete mitochondrial genome of *T. callipaeda* (GenBank Accession Number AP017700) that was published as part of the 50 Helminth Genomes initiative (https://www.sanger.ac.uk/science/collaboration/50HGP) and 99.8% (498/499 nt) identical to haplotype h1 (GenBank Accession Number AM042549), the single haplotype circulating in Europe (Otranto et al. 2005). The single nucleotide difference with AM042549 was located in the sequence of the forward primer used in that study.

## Discussion

Since its first detection in Italy in the 1980s, *T. callipaeda* has spread across Europe, with recent cases of autochthonous thelaziosis in carnivores from Bulgaria (Colella et al. 2016), Slovakia (Cabanova et al. 2017), Hungary (Farkas et al. 2018), Moldova (Dumitrache et al. 2019) and Austria (Hodzic et al. 2019), as well as several reported human cases (Paradzik et al. 2016; Tasic-Otasevic et al. 2016; Dolff et al. 2020). Important factors in the expansion of canine thelaziosis include the travelling of infected dogs to/from endemic regions and the presence of a sylvatic cycle mainly maintained by foxes (Otranto et al. 2009; do Vale et al. 2019).

Previous reports from Germany concerned a dog that had recently visited Italy (Hermosilla et al. 2004), two dogs and a cat with a recent presence in endemic countries (Silva et al. 2020) and a probable autochthonous case of a dog in southwestern Germany, near the French border (Magnis et al. 2010). An interesting aspect of the case presented here is the fact that the dog lived in northern Germany and had not entered regions known to be endemic for *T. callipaeda*. Her southernmost visit had been a daytrip to the German-French border area near Saarbrücken in April 2017. Although this region is not known to be endemic for *T. callipaeda*, it cannot be completely excluded that the initial infection occurred here, as the longevity of adult nematodes was reported to be more than 1 year (Faust 1928). However, the fact that the conjunctivitis symptoms occurred early August 2019 speaks more in favour of a local transmission in northern Germany. *Phortica variegata*, the intermediate host of *T. callipaeda*, is present in Germany (Bächli 2019), and a recent ecological niche model showed that large areas of Europe, including Lower Saxony where the dog lived, are suitable for the development of *P. variegata* (Palfreyman et al. 2018). The lachryphagous activity of male *P. variegata* was shown to be associated with temperature, with 7 to 11 °C being required as a minimum temperature for the start of activity (Pombi et al. 2020). In Lower Saxony, daily average temperatures above 7 °C were reported from May to October 2019 (wetterkontor.de 2020), suggesting that this will not form a limitation for the transmission of *T. callipaeda*. However, more research, for instance, into the local activity of *P. variegata* and the prevalence of *T. callipaeda* in domestic and wild carnivores in Germany would be required to confirm the presence of a local transmission cycle.

This case report shows that veterinarians in northern Europe should be aware of canine thelaziosis, as it presents an emerging vector-borne disease with zoonotic implications in Europe.
